# Time-lapse imaging of cells in spatially fractionated X-ray fields using a mini beam as an alternative to accelerator-based sub-millimeter beams

**DOI:** 10.1093/jrr/rraf020

**Published:** 2025-05-11

**Authors:** Kiichi Kaminaga, Hisanori Fukunaga, Eri Hirose, Ritsuko Watanabe, Keiji Suzuki, Kevin M Prise, Akinari Yokoya

**Affiliations:** Institute for Quantum Life Science, National Institutes for Quantum Science and Technology, 4-9-1 Anagawa, Inage, Chiba 263-8555, Japan; Faculty of Health Sciences, Hokkaido University, N12 W5 Kita-ku, Sapporo 060-0812, Japan; Center for Environmental and Health Sciences, Hokkaido University, N12 W7 Kita-ku, Sapporo 060-0812, Japan; Sector of Nuclear Fuel, Decommissioning and Waste Management Technology Development, Japan Atomic Energy Agency, 4-49 Muramatsu, Tokai-mura, Naka-gun, Ibaraki 319-1112, Japan; Institute for Quantum Life Science, National Institutes for Quantum Science and Technology, 4-9-1 Anagawa, Inage, Chiba 263-8555, Japan; Department of Radiation Medical Sciences, Atomic Bomb Disease Institute, Nagasaki University, 1-12-4 Sakamoto, Nagasaki 852-8523, Japan; Patrick G Johnstone Centre for Cancer Research, Queen’s University Belfast, 97 Lisburn Road, Belfast BT9 7AE, UK; Institute for Quantum Life Science, National Institutes for Quantum Science and Technology, 4-9-1 Anagawa, Inage, Chiba 263-8555, Japan; Graduate School of Science and Engineering, Ibaraki University, 2-1-1 Bunkyo, Mito, Ibaraki 310-8512, Japan

**Keywords:** collimator, FUCCI, HeLa cell, sub-millimeter beam, non-uniform radiation fields

## Abstract

Due to the limited number of accelerator-based X-ray facilities worldwide that provide beams with an adjustable size, their application for radiobiological research purposes has been restricted. Thus, the development of alternative methods is of technical importance for investigating cell/tissue responses in spatially non-uniform radiation fields. In this study, we performed mini beam irradiation of cells using a lead (Pb) sub-milli-collimator as an alternative method to sub-millimeter beams. Also, we employed human cervical carcinoma HeLa cells and hTERT-immortalized fibroblast BJ-1 cells that express fluorescence ubiquitination-based cell-cycle indicators (FUCCI). Time-lapse imaging revealed differences in the behavior of HeLa and BJ-1 cells in spatially heterogeneous radiation fields; in the case of HeLa cells, G2/M phase-arrested cells in the cell population were clearly observed, distinguishing irradiated from non-irradiated cells at the sub-millimeter scale level. Our findings indicate that FUCCI can be useful as a biological dose indicator, depending on cell type, and Pb sub-milli-collimators show potential as a possible alternative to accelerator-based X-ray sub-millimeter beams for radiobiological research. The use of the collimators, unlike beamtime experiments in synchrotron facilities with the approval of the committee, is highly versatile and may be beneficial in preliminary studies in a normal laboratory environment.

## INTRODUCTION

Radiation-related effects at the tissue level are generally dose dependent; nevertheless, the tissue response can vary markedly depending on whether the radiation exposure is uniform or non-uniform. Furthermore, an important element of any non-uniform exposure is the unpredictability of the projected dose–response relationship based on standard radiobiological DNA damage and repair models and dose–volume histogram interpretations, as the underlying mechanism remains unclear [1]. One of the crucial factors contributing to this unpredictability is the tissue-sparing effect (TSE), which refers to the acquisition of radiation tolerance at the tissue level caused by exposure to spatially fractionated radiation [2]. Since the first observation of TSE in radiotherapy was reported using a grid pattern irradiation in the 1900s [3], several studies have been performed in terms of clinical application of spatially restricted narrow radiation beams [4, 5]. These pioneer works were succeeded by microbeam radiotherapy (MRT) using various radiation sources [6–14]. To date, accelerator-based X-ray or particle beams with an adjustable size, particularly microbeams equipped with a single-cell irradiation apparatus, have been developed as popular tools to irradiate individual, micron-sized samples or portions of samples [15], for investigating not only TSE but also ‘non-targeted effects’ such as abscopal effects, clastogenic effects and bystander effects [16].

Recently, sub-millimeter beams have been also applied to provide spatially fractionated technique in radiation therapy known as a mini beam using synchrotron X-rays [17, 18], proton beams [19–21] or ion beams [22, 23]. Although demands of such mini-beam irradiations have been expanded year by year, there are a limited number of beam apparatuses with an adjustable size in the accelerator research facilities, especially those capable of X-ray irradiation. Therefore, the development of alternative methods has long been expected for radiobiological research purposes.

In this study, we used a conventional lead (Pb)-shielding collimator providing sub-millimeter width X-ray beam (hereafter, mini beam) to realize the spatially non-uniform radiation exposure conditions using a laboratory X-ray source. In addition to the collimator, we employed human cervical cell line HeLa cells expressing fluorescence ubiquitination-based cell-cycle indicators (FUCCI). The FUCCI technique was developed to augment distinct visualization of cell-cycle phases by fluorescence microscopy [24], suggesting the possibility that it would be useful to detect the non-targeted effects of radiation on cell cycle and mobility [25].

## METHODS

### Cell culture

A subline of the HeLa-FUCCI cells, RCB2812 HeLa.S-FUCCI [26], was provided by the RIKEN BioResource Center in Japan. The cells were cultured in Dulbecco’s modified Eagle’s medium (Wako Pure Chemicals, Osaka, Japan), containing 10% fetal bovine serum (Biological Industries, Kibbutz Beit-Haemek, Israel) and 1% antibiotic–antimycotic (Life Technologies, Carlsbad, CA, USA), in a humidified incubator maintained at 37°C in an atmosphere with 95% air and 5% CO_2_. The day before irradiation, logarithmically growing cells (2 × 10^5^cells) were seeded into 35-mm-φ cell culture dishes (Falcon), and the medium was changed after X-irradiation to start time-lapse imaging.

As previously described [25], the HeLa-FUCCI cells were able to bypass the G1 checkpoint, whereas the G2/M checkpoint was functional. In fact, a previous study reported that the durations of the red phase do not change even when HeLa-FUCCI cells are irradiated with up to 10 Gy [27]. The G1/S checkpoint governed by p53 activated through ATM and its downstream factor, Chk2, does not function properly in HeLa cells [28]. In contrast, the G2/M checkpoint could work via a pathway through which cell-cycle checkpoint factors, Chk1 and Cdc25, are activated by ATR [29]. Thus, there was remarkable G2/M cell-cycle arrest in the exposed HeLa-FUCCI cells, supporting that the release from radiation-induced cell-cycle arrest requires cellular processes that presumably sustain Chk1’s inhibition pathway of Cdc2/Cyclin B phosphorylation by Cdc25 [30].

We also used hTERT-immortalized human normal fibroblast BJ-1 cells with wild-type p53 function that express FUCCI (BJ-1-hTERT-FUCCI cells). Unlike epithelial cells, mesenchymal cells exist in the extracellular matrix, unbound to their surroundings, and are characterized by their motility. The cells were cultured in the same culture medium and conditions as described above. The cell-cycle checkpoint is functioning normally in these cells.

### X-ray settings

The cells were exposed to a conventional X-rays through the lid of a culture dish using an X-ray generator with a W-target (Softex, Kanagawa, Japan) operated at a tube voltage of 150 kV and a tube current of 4.1 mA [31]. The samples were exposed to X-rays from the tungsten anode. The energy spectrum from the X-ray machine provided by the manufacturer showed that there are characteristic X-ray peaks ~60 keV ascribed to the tungsten target of X-ray tube. The 0.2-mm aluminum filter was applied to filter out the X-rays <7 keV. We measured the attenuation factor of air at the sample position using an ionizing chamber. The obtained half value of air layer was 10.9 cm at 25°C, 1 atm, indicating that the effective energy of the X-rays was ~45 keV, which was slightly lower than those of characteristic X-ray peaks (around 60 keV) from the tungsten target because of bremsstrahlung in the lower energy region. The X-ray field was ~10 cm in diameter at the sample position. The distance from X-ray window to the sample surface of the cells in the culture dish was 29.6 cm.

Prior to irradiation experiments, we measured the dose at the sample position by Fricke dosimetry method with a Fricke solution with a depth of a few millimeters based on the assumption that the cultured cell samples were approximated as that of water. We periodically checked whether the dose was properly delivered with an ionization chamber by detecting an X-ray flux (C/kg) used as secondary standard. The Fricke solution was exposed to X-rays for designated time (e.g. 5 or 10 min) without any collimators. The dose rate was determined with the measured dose and exposure period to be 1 Gy/min, and the total doses of the X-rays absorbed by the cells were set to 10 Gy at the surface of the collimator. In order to simulate the microenvironment of irradiated tissue, the confluence level before irradiation was ~90–100%.

### Spatially fractionated irradiation settings using a sub-milli-slit

**Fig. 1:**
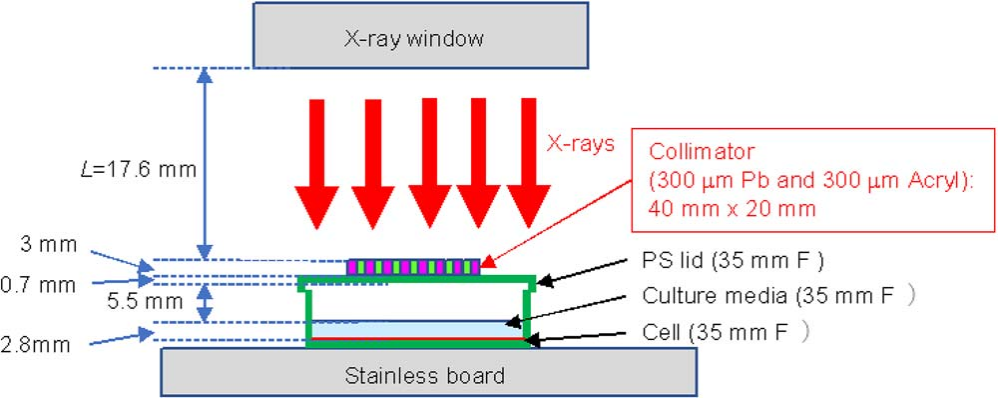
Irradiation settings. Schematic representation of the sub-milli-slit-modulated irradiation field. Cells were irradiated in a 35-mm-diameter dish (Falcon 35 mm Easy-Grip dish) below the lead (Pb) collimator.

Spatially fractionated irradiation settings were performed using the X-ray source and a collimator consisting of lead sheets of 300-μm thickness. As previously described [31], Pb sheets with a size of 3 (wide) × 15 (height) × 0.3 (thickness) mm (Nirako, Tokyo, Japan) and acrylic plates of the same thickness through which X-rays are transmitted were alternately layered and set downstream of the X-ray source to make 300-μm slit-modulated X-ray beams (600 μm for peak-to-peak). Figure 1 shows the geometry of irradiation setup for non-uniform exposure using the collimator.

Prior to sample irradiation, the dose profiles were estimated by Gafchromatic XR-RV3 radiochromic film (Ashland Inc., Covington, KY, USA, hereafter Gafchromatic film). The film was placed directly below the collimator to obtain the picture of a color change profile by the X-ray exposure (see Fig. 3A). The color-changing of the films irradiated with various X-ray doses without the sub-milli-slit were also measured under bright-field illumination using a Leica stereomicroscope (M205C) to obtain a standard curve to estimate the dose in the sub-millimeter range. The acquired images were analyzed for brightness using the line profile function in ImageJ (NIH, 1.54p). A calibration curve was obtained as a fitting curve of the brightness values plotted against doses as shown in the supplemental figure (Fig. S1).

**Fig. 2:**
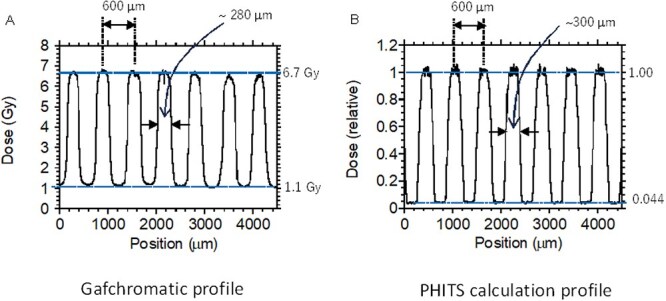
Dose profile of the modulated irradiation field with a slit size of 300-μm width. The dose distribution was confirmed using a Gafchromic XR-RV3 radiochromic film (A) and PHITS code (B). The measurement results show that the doses in the valley area are relatively higher than the theoretical ones.

The collimator image showed a clear difference in the dose distribution, as shown in Fig. 2A, between the peaks and valleys reflecting the slit width of 300 μm (600 μm for peak-to-peak). The irradiated area of the sub-milli-slit profile taken by the Gafchromatic film showed that the dose delivered in the irradiated area was ~6.7 Gy, which was less than the expected value of 10 Gy (see above) because of significant attenuation by the acrylic plate in the collimator. On the other hand, the non-irradiated areas were also delivered a certain dose of an average of 1.1 Gy, presumably because of backscattering components from the stainless sample tray as well as scattered radiations from a plastic lid of the dish, etc. Thus, the average value of the peak-to-valley dose ratio (PVDR) was 0.16. The relative error of the peak and valley value were 0.017 (1.7%) and 0.007 (0.07%), and there is a clear difference in the dose distribution between the peaks and valleys (Fig. 2A). These estimations indicated that, although not as accurate as microbeams, sub-milli-slit irradiation using the collimator was useful enough for radiobiological experiments.

The dose profile was also complementally estimated by the Monte Carlo code of Particle and Heavy Ion Transport Code System (PHITS ver. 3031) [32]. The PHITS simulation was performed using the geometry shown in Fig. 1. The X-rays with the provided spectra were set to almost perpendicular to the collimator and only slightly divergent (0.5-degree solid angle). The distance between the X-ray window and the collimator surface, *L*, is 50 mm, which is much larger than the experimental condition to save the simulation time. The photons and secondary electrons were simulated in the calculation, where the cut-off energies for photons and electrons were set to be 1.0 keV. Figure 2B shows the simulated dose profile by the PHITS calculation. The peaks and valleys reflecting the slit width of 300 μm, 600 μm for peak-to-peak, were confirmed. The dose was normalized by the average dose for the peak area. The profile reproduced roughly the dose distribution on the Gafchromatic film. The PVDR value was, however, ~0.05, which was significantly smaller than that obtained by the Gafchromatic films (0.16 as described above). Although we considered the backscattering component from the stainless sample tray in the PHITS calculation, diffusive X-ray trajectory, not exactly parallelism beam, and scattered radiations from a plastic lid of the dish, air layer and culture medium, and diffuse reflections from sidewalls, the ceiling board in the irradiation chamber also needs to be included in the calculation to reproduce the Gafchromatic profiles exactly.

### FUCCI time-lapse imaging

As previously described [25], to visualize red (570 nm) and green (505 nm) fluorescence emissions from the cell nuclei, a BZ-X700 fluorescence microscope (KEYENCE, Osaka, Japan) equipped with an automatic filter wheel was employed. The cultured samples were observed with 4× and 20× magnification objective lenses to capture FUCCI-fluorescent and bright-field images every hour for 24 h. The FUCCI signal fluorescence was emitted only from cell nucleus. The boundary of a cell was confirmed by overlying the fluorescent image with the bright-field image.

Geminin is ubiquitinated during the M and G1 phases and degraded by proteasome, while Cdc10-dependent transcript 1 (Cdt1) is ubiquitinated during the S and G2 phases and degraded by proteasome. Thus, by combining Geminin expressed in the S/G2 phases and Cdt1 expressed in the G1 phase with green and red fluorescent proteins, respectively, the nuclei of HeLa-FUCCI and BJ-1-hTERT-FUCCI cells in the G1 phase are shown in red, and those in the S/G2 phases are green under a fluorescence microscope. The early S phase, when the expression of both overlaps, is yellow. The green and red fluorescence intensities of HeLa-Fucci cells and BJ-1 hTERT-Fucci cells were analyzed by using the line profile function of ImageJ. The fluorescence ratios were calculated based on the intensities as shown in Fig. 7 (see below).

## RESULTS

### HeLa-FUCCI cells following exposure to spatially fractionated X-rays

**Fig. 3:**
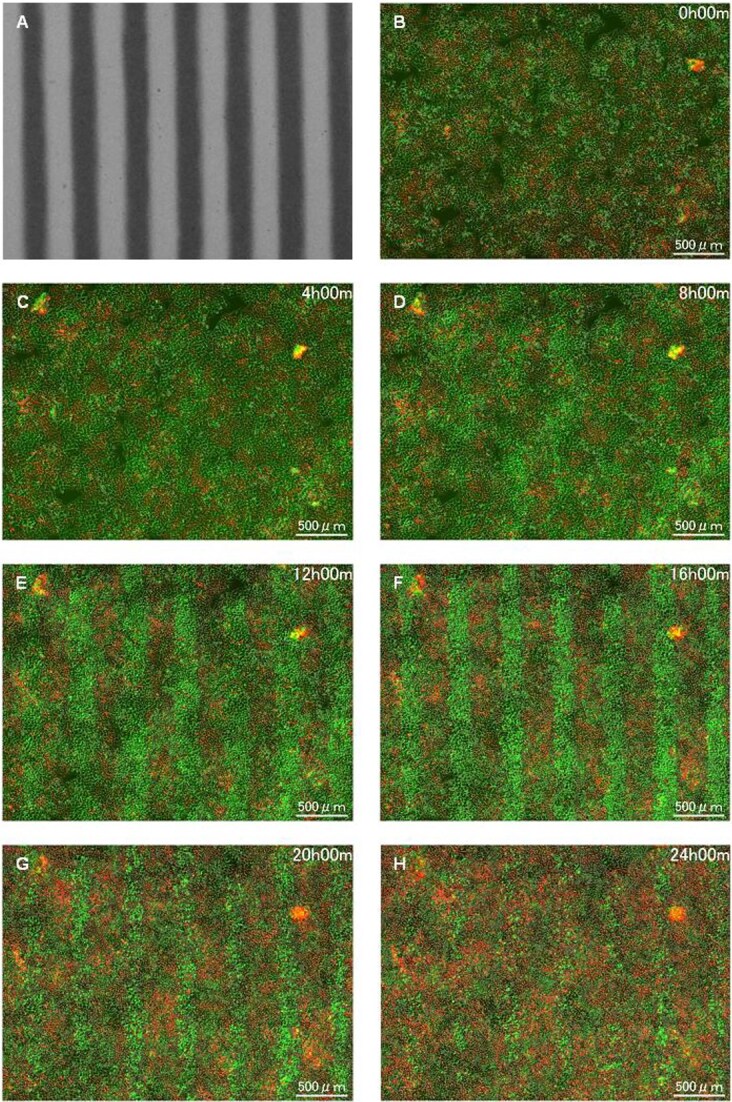
Time-lapse imaging on HeLa-FUCCI cells following exposure to spatially fractionated X-rays. (A) Dose profiles of the 300-μm-slit field using a Gafchromic XR-RV3 radiochromic film. (B–H) Representative fluorescent images of HeLa-FUCCI cells observed 4× magnification objective lens at 0 (B), 4 (C), 8 (D), 12 (E), 16 (F), 20 (G) and 24 h following exposure to the 300-μm-slit X-rays (H). Cell nuclei are indicated by the red, yellow, green and colorless regions depending on their cell-cycle phase, G1, G1/S, S/G2 and M, respectively. Scale bar—500 μm. In this study, the reproducibility of imaging results was confirmed from three or more independent experiments performed on different days.

**Fig. 4:**
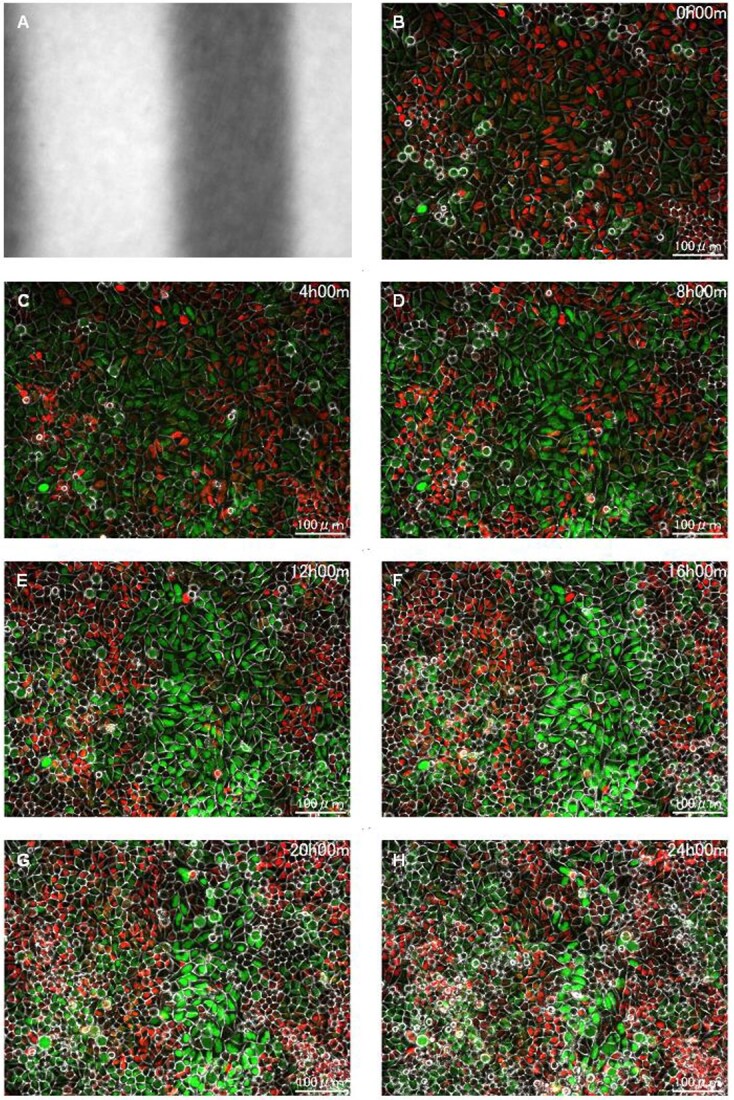
Magnified time-lapse imaging on HeLa-FUCCI cells following exposure to spatially fractionated X-rays. (A) Dose profiles of the 300-μm-slit field using a Gafchromic XR-RV3 radiochromic film. (B–H) Representative magnified fluorescent images of HeLa-FUCCI cells observed 20× magnification objective lens at 0 (B), 4 (C), 8 (D), 12 (E), 16 (F), 20 (G) and 24 h following exposure to the 300-μm-slit X-rays (H). Cell nuclei are indicated by the red, yellow, green and colorless regions depending on their cell-cycle phase, G1, G1/S, S/G2 and M, respectively. Scale bar—100 μm.

Figures 3 and 4 illustrate the HeLa-FUCCI cells following exposure to spatially fractionated 6.7-Gy X-rays. Figures 3A and 4A are dose profiles showing the irradiation range. In the 4× magnification image, cells undergoing cell-cycle arrest were clearly seen in the form of slits, as there was little cell migration within and outside the irradiation area (Fig. 3B–H). The striped pattern was pronounced between 12 and 16 h. In the 20× magnification (Fig. 4B–H), cells undergoing cell-cycle arrest remain in the irradiation field. These results demonstrate that the radiation effect remained in the HeLa-FUCCI cells in the irradiated area. The reproducibility of imaging results was confirmed from three or more independent experiments performed on different days. This result is also similar to our previous study conducted using synchrotron X-ray microbeams [25], ensuring the reproducibility.

### BJ-1-hTERT-FUCCI cells following exposure to spatially fractionated X-rays

**Fig. 5:**
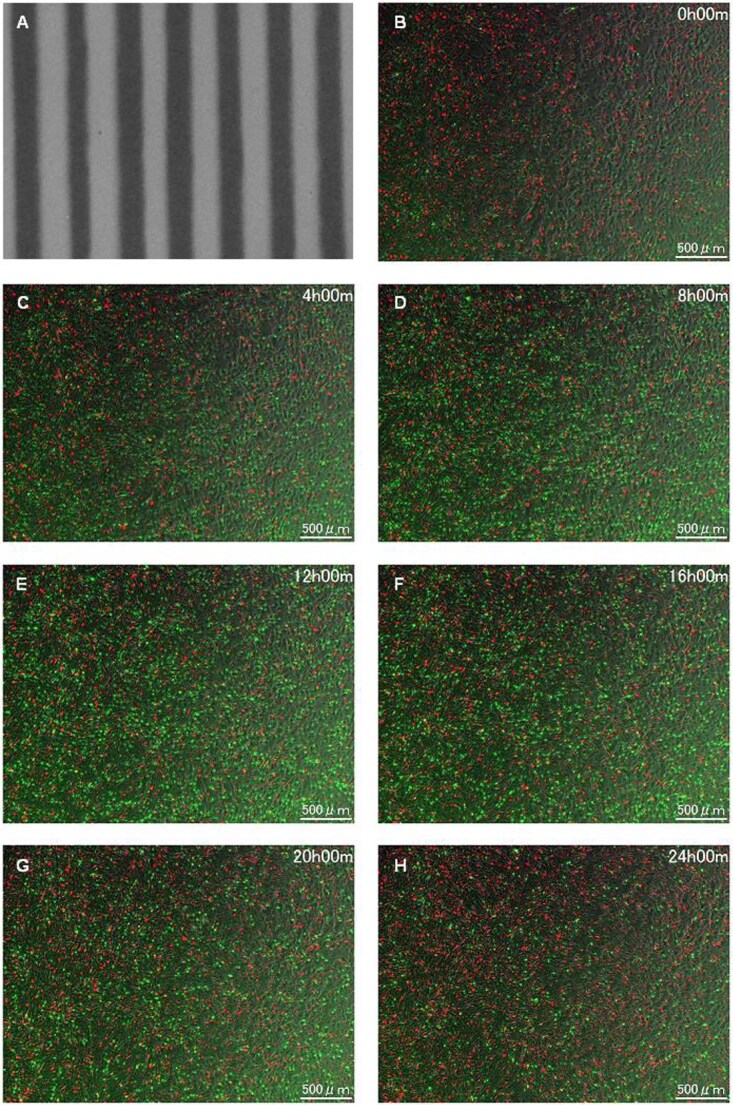
Time-lapse imaging on BJ-1-hTERT-FUCCI cells following exposure to spatially fractionated X-rays. (A) Dose profiles of the 300-μm-slit field using a Gafchromic XR-RV3 radiochromic film. (B–H) Representative fluorescent images of BJ-1-hTERT-FUCCI cells observed 4× magnification objective lens at 0 (B), 4 (C), 8 (D), 12 (E), 16 (F), 20 (G) and 24 h following exposure to the 300-μm-slit X-rays (H). Cell nuclei are indicated by the red, yellow, green and colorless regions depending on their cell-cycle phase, G1, G1/S, S/G2 and M, respectively. Scale bar—500 μm. In this study, the reproducibility of imaging results was confirmed from three or more independent experiments performed on different days.

**Fig. 6:**
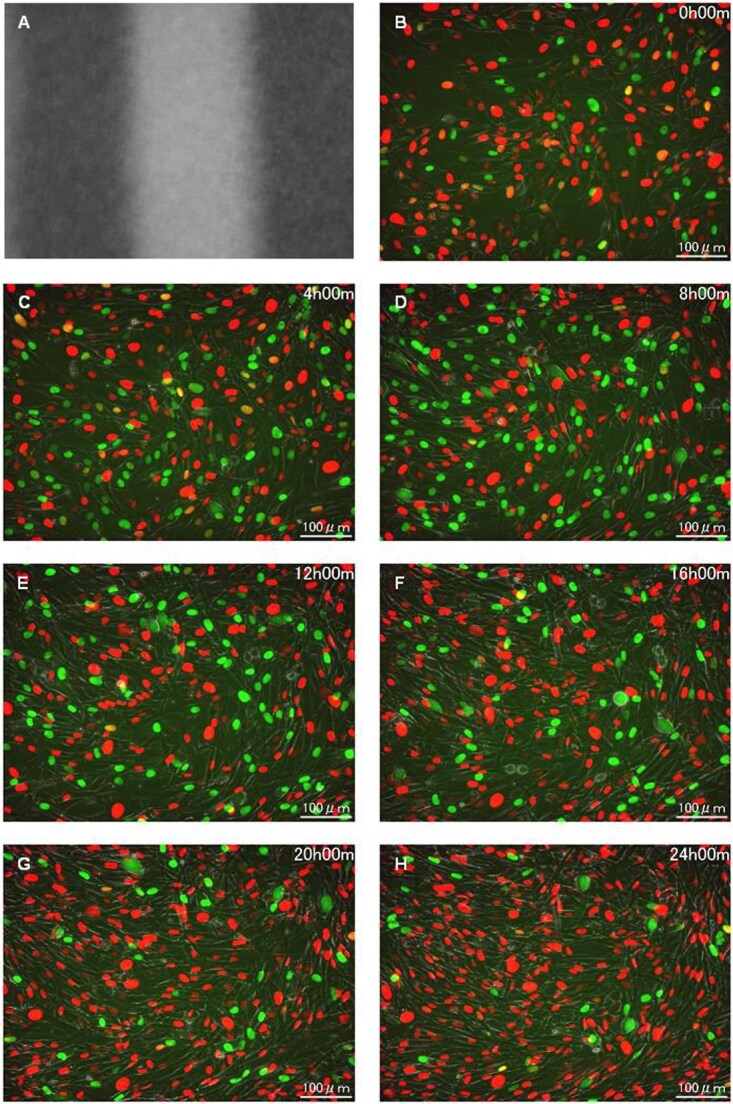
Magnified time-lapse imaging on BJ-1-hTERT-FUCCI cells following exposure to spatially fractionated X-rays. (A) Dose profiles of the 300-μm-slit field using a Gafchromic XR-RV3 radiochromic film. (B–H) Representative magnified fluorescent images of BJ-1-hTERT-FUCCI cells observed 20× magnification objective lens at 0 (B), 4 (C), 8 (D), 12 (E), 16 (F), 20 (G) and 24 h following exposure to the 300-μm-slit X-rays (H). Cell nuclei are indicated by the red, yellow, green and colorless regions depending on their cell-cycle phase, G1, G1/S, S/G2 and M, respectively. Scale bar—100 μm.

Figures 5 and 6 show the cell-cycle arrest among the BJ-1-hTERT-FUCCI cells following exposure to spatially fractionated 6.7-Gy X-rays. Figures 5A and 6A are dose profiles showing the irradiation range. Notably, the 4× image showed no difference in radiation effects both inside and outside the irradiation range (Fig. 5B–H). The cells exhibiting cell-cycle arrest after radiation exposure were not confined to the irradiated area (Fig. 6B–H). In contrast to the HeLa-FUCCI cells, interestingly, the boundary between the irradiated and non-irradiated area was not distinct, and there appeared to be no difference in the cell-cycle distribution of cells in each area. In this study, it was not possible to track each individual cell in this time-lapse condition due to the high confluence level.

## DISCUSSION

### Sub-milli-collimator irradiation as an alternative sub-milli beam method for radiation biology

In this study, time-lapse imaging revealed that sub-milli-collimators have potential as an alternative to accelerator-based X-ray beams with adjustable sizes. The use of sub-milli beams for radiobiological research purposes has long been limited due to the small number of facilities and finite beamtime. Further development of clever shielding techniques, such as lead ‘micro-collimators’ as an alternative to accelerator-based microbeams, will expand the research on such heterogeneous radiation exposure effects. The accelerator-based microbeam systems typically irradiate single (or multi) cells in discrete positions in a fashion of step-by-step shooting. Thus, one needs longer irradiation time for a larger number of cells and the condition of the initially exposed cells would be different from those of finally exposed cells. Contrary to this, the micro-collimated broad field delivers dose to each exposed area simultaneously, thus preventing cells from variation of exposure times. Furthermore, collimators are more versatile than microbeams because they can be used in a normal laboratory environment and are expected to work with a variety of devices.

There are technical limitations of the collimator approach. One of the important issues is the higher dose at the valley area of the sub-milli beams, compared to that using synchrotron-generated mini beams. One possible reason is that the beam spread and scattering would occur in the space between the slit and the sample, namely, the dish lid, air layer and culture medium. Thus, to make the slit irradiation more effective in the future, it may be necessary to (1) make the slit and sample surface as close as possible, and (2) shield the beam line and sample with lead to prevent scattering from the surroundings.

The establishment of a spatially non-uniform irradiation method using the sub-milli-slit irradiation, as shown in the current study, is expected to be a driving force for further development of MRT research, which has been carried out only at synchrotron radiation facilities. In our previous study, we performed sub-milli-slit irradiation of cultured testes using the sub-milli collimator [31]. However, it was not clear whether such an irradiation method would allow examination at the cellular level. This study is first to indicate that the use of collimators is useful for analyzing cellular responses in spatially fractionated radiation fields.

### FUCCI’s potential for dosimetric assessment

In a sub-milli-slit-modulated radiation field, HeLa-FUCCI cells showed the presence of cell-cycle arrest cells confined to the irradiation field, while BJ-1-hTERT-FUCCI cells showed no clear boundary between the non-irradiated and irradiated field in which the cell-cycle distributions were similar. The preliminary results presented in this study suggest that cell behavior in spatially inhomogeneous radiation fields may differ among cell types. In the future, more detailed measurement results will be required for novel biological data analysis, e.g. time-lapse conditions, verification of cell migration for each cell type and statistical treatment of data.

**Fig. 7:**
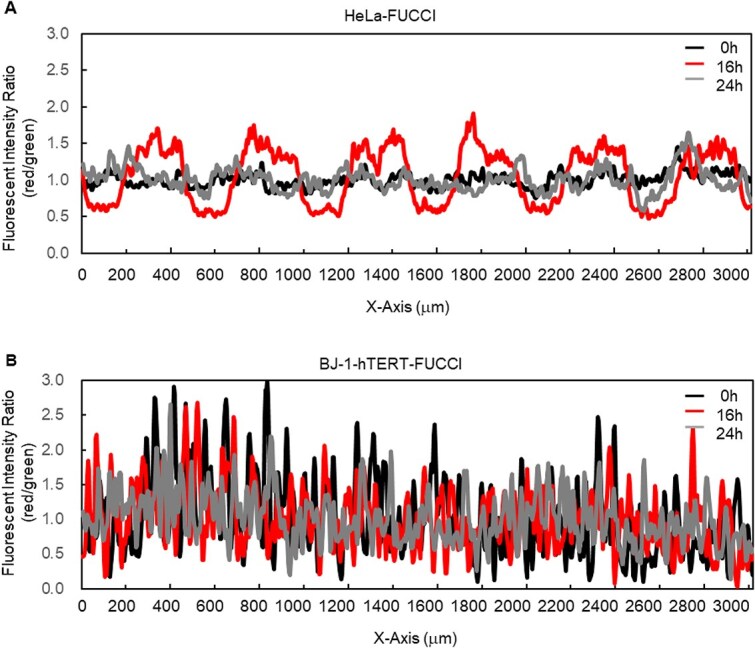
Relative quantitative changes in fluorescence intensity ratio for HeLa-FUCCI and BJ-1-hTERT-FUCCI cells. (A) Plot the ratio of the relative value of each fluorescence (red/green) for HeLa-FUCCI cells. (B) Plot the ratio of the relative value of each fluorescence (red/green) for BJ-1-hTERT-FUCCI cells. The average fluorescence intensities of a 3 (width) × 1 (height) mm region in red and green fluorescence images at 0, 16 and 24 h were calculated, then each ratio (red/green) of the relative intensity values was plotted.

We also noticed that HeLa-FUCCI cells are a possible indicator of irradiation coverage. Unlike BJ-1-hTERT cells, HeLa cells show distinct G2/M arrests and adhesiveness, making HeLa-FUCCI cells suitable as short-term biomarker for dosimetric assessment in sub-millimeter region. As shown in Fig. 7, 16 h after irradiation, HeLa-FUCCI cells clearly showed the irradiated and non-irradiated areas. Thus, the FUCCI technique is useful for identifying exposed cells, suggesting potential applications for the dosimetric studies. One of the technical limitations of this study is that we only analyze the behavior of *in vitro* HeLa and BJ-1-hTERT cells. Further investigation with other human cell lines is required to validate our findings.

### Cell migration as a possible damage control mechanism at the tissue level

Cell migration is crucial in the wound healing process; when a tissue is injured, fibroblasts get activated and differentiate into myofibroblasts, which generate large contractions and actively produce extracellular matrix proteins to facilitate wound closure [33]. The different response at the tissue level when comparing the case of uniform exposure of all cells in the irradiated area with the case of spatially fractionated exposure may be due to the dilution of radiation effects by the migration of some cells, such as fibroblasts. While dampening the acute effects at the tissue level, it also has the potential to spread the radiation-induced consequences of both the targeted and non-targeted effects, outside the irradiation area through cell migration of irradiated cells. From the viewpoint of radiotherapy and radiation protection, it is necessary to analyze how the radiation response is diluted or diffused in the situation where non-irradiated and irradiated cells are mixed. For this purpose, it will also be necessary to investigate the cellular behavior of each cell within and without the irradiated areas, as suggested by the results of this study. In particular, it is necessary to clarify the significance of the behavior of cells before cell death occurs.

An efficient way to mitigate the radiation-induced effects at the tissue level would be to promote or inhibit the activity of cell migration. For instance, in radiotherapy, suppressing the migration of tumor cells and keeping them within the planning targeted volume as much as possible could potentially have a significant impact on the response rate. In fact, several studies have indicated a link between cell migration activity and radiation effects for cancer therapy [34]. Radiobiological studies using lead sub-milli collimators as an alternative to adjustable size system of accelerator-based X-ray beams could contribute to the development of this field.

## Supplementary Material

supplementary_not_blind_rraf020

BJ-1_FUCCI_movie_rraf020

HeLa_FUCCI_movie_rraf020

## Data Availability

The data underlying this article will be shared on reasonable request to the corresponding author.
