# Supplementary material for: Time-lapse imaging of cells in spatially fractionated X-ray fields using a mini beam as an alternative to accelerator-based sub-millimeter beams
Source: J Radiat Res. 2025 May 11;66(3):318–28. doi: 10.1093/jrr/rraf020 (PMC12100484; doi:10.1093/jrr/rraf020)
Supplement: supplementary_not_blind_rraf020 [file supplementary_not_blind_rraf020.docx]

**Time‒lapse imaging of cells in spatially fractionated X‒ray fields using a mini beam as an alternative to accelerator‒based microbeams**

Kiichi Kaminaga, Hisanori Fukunaga^*^, Eri Hirose, Ritsuko Watanabe, Keiji Suzuki,

Kevin M. Prise, and Akinari Yokoya^*^

Supplementary data:

Supplementary_FigureS1

Supplementary_Movie1

**
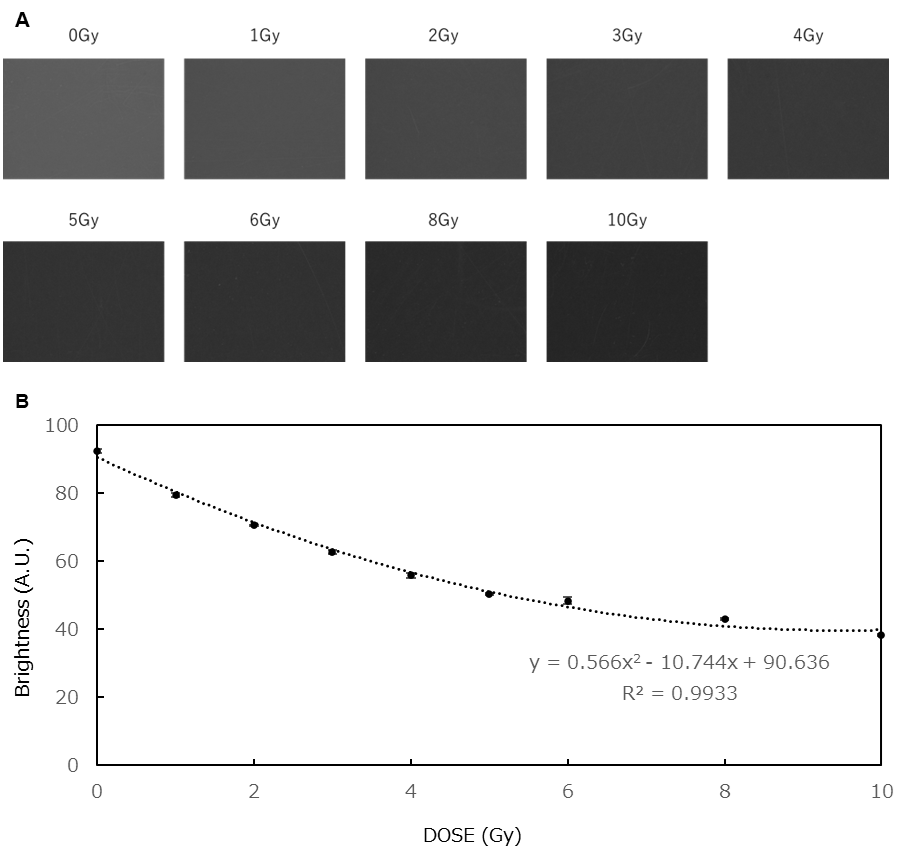
Figure S1.** Radiochromic film dosimetry settings. (A) Typical images of radiochromic film after 0-10 Gy irradiation. (B) Calibration curve for dosimetry.
